# Annotations

**Published:** 1893-07-08

**Authors:** 


### ANNOTATIONS.

**The "Drink-Crave Cure."**

Four circulars have been sent to us commending general consideration a "cure" for what is called the "drink crave." Every conscientious medical man would welcome a cure for drunkenness; and provided it was real and actual would not too nicely scrutinise the source from which it emanated. However prejudiced our medical ancestors may have been, we, their professional descendants, are minded to lay fast hold of "cures" and to stick to them, wherever they may happen to come from. The only condition we insist upon is that they shall be *bona fide*. The particular "cure" now offered to the public presents very sorry credentials. It has been experimented with by a number of persons at Birmingham, not one of whom appears to have been a medical man—most of them seem to have been Sunday school teachers, or Temperance Society officials. To this we should not have particularly objected—because anybody can see whether a person is drunk or sober—it the so-called experiments had been of any value. But one fact alone is sufficient to show their untrustworthiness, not to say their childishness. The experiments were begun on the twenty-third of May, and the circular accompanying the committee's report bears date the twentieth of June. So that the whole business appears to have occupied less than a month. There is no need for discussion here. The futility of the performance demonstrates itself. The truth is that any third year's medical student can tell the experimenters of a dozen drugs, any one of which will nauseate a drunkard and "cure" him of his drink crave for at least a month. One would like to know the average age of the members of the Birmingham committee. Surely most of them must be still in the "pinafore" stage! We did not give Birmingham credit for so much gullibility.

**A Unique City Company.**

The Worshipful Company of Plumbers is one of the smallest of the City companies, and one of the least well endowed. But though the company is so small it has an extraordinary spirit. For the last fifteen or twenty years it has had the generous ambition to revolutionise the practical sanitation of the country, and to bring it thoroughly abreast of the most scientific requirements of modern times. In order to understand the courage and far-sighted intelligence of the heads of the company, we have to consider what such a purpose as is here indicated involves. It involves nothing less than the scientific and practical education, the registration, and the adequate control of every member of the plumbing art and business throughout the country. This is a vast scheme; and what is more, it is in the way of being realised. The Plumbers' Company have been wise in their generation. They have gone soberly to work, using the modern methods of instruction and persuasion. The card of compulsion they have quietly dropped on the floor, at any rate until the time for just and legitimate compulsion should arrive. Gibbon said that "persuasion is the resource of the feeble;" the Plumbers' Company say that persuasion is the resource of the far-sighted when they have but limited resources and a great purpose. What has the company already done towards making the sanitation of the country perfect, and annihilating those innumerable forms of disease which have their origins in insanitary conditions? It has, in the first place, satisfied all the plumbers and sanitarians throughout England, Ireland, Scotland, and Wales that its objects are entirely good, and ought to be supported—a most statesmanlike achievement. It has established centres for the technical education of plumbers throughout the country, and appointed competent teachers. It has created an examining body whose examinations are trusted, and whose certificates are valued by those who best understand the subject. And, at the present moment, it is piloting a Bill through the House of Commons to make the education and voluntary registration of professed plumbers easy throughout the whole of the United Kingdom. This is a brief outline, but a truly worthy record. The Lord Mayor, Mr. Stuart Knill, is the present master of the company, and a most enthusiastic technical educator. The past master and present warden, Mr. W. H. Bishop, is equally enthusiastic, and, though a stockbroker, is a most ardent sanitarian and plumber. All this work, done for the most part out of sight by the Plumbers' Company, demands some public recognition; and the recognition which the heads of the company would value most would be the speedy passing of the Registration Bill, now before Parliament, into law. All Members of Parliament who respect patience, pluck, disinterestedness, and worthy public aims should see to it that the Bill, which is universally approved, is transferred to the Statute Book.

**Summer Cobwebs and the Broom.**

The spiders of the brain spin with extraordinary rapidity as July progresses and August draws near. We are conscious of obfuscations, of disproportions of all kinds. The "sweetness" of the most reasonable temper tends to become acidulated; the "light" of the clearest intelligence is clouded, and gives distorted presentations even of common things. Under these conditions the tasks of life swell out and assume the sizes and shapes of impossibility; the conscious capacity on the other hand shrinks and halts until we begin to think that there is nothing for it but to surrender and let other hands take up that which has proved too much for ourselves. It is all nonsense, fellow-workers! We want our holiday, that is all. The annual holiday is part of the contract in strenuous civilised life. It is as real a necessary as a house or a winter overcoat. For we are bound by every consideration of honour and self-respect to do our very utmost in life's long cricket match. We must each secure a victory of some sort. It may not be quite the victory we set out to win: the throne of the Archbishop of Canterbury, the rosebed of the Prime Minister, the Woolsack, the presidency of the College of Physicians, or the civic grandeur of the Lord Mayor. But a victory of some kind each of us owes it to himself to win. And the victory is only possible to him who patiently and loyally, as well as strenuously, observes the first conditions of the contest. One of the conditions is a good, breathing, life-renewing summer holiday. We must take it, even though some of us may say in these bad times that we cannot afford it. The truth is, we cannot afford not to take it. Somebody has told us to "live by the day." That is philosophy most sound, but it is philosophy in the alphabet only. We must live by the year also; and by the life-time. Granted that we are not so far forward this summer as a year ago we hoped we should be; granted that some of us may even have suffered a temporary reverse: the life that is yet before us demands our very best powers of brain, of will, of physique, of morale. Let us unbend the bow then at the reasonable time; let us unbend it thoroughly. A month's change, a month's fellowship with sea or country, with life from which strenuousness has ceased for a time, will make new men and women of us all. Let us every one put down the tools of our trade as soon as may be, and give ourselves the schoolboy's perennial delight of a good, long holiday.

